# Overview of *Helicobacter pylori* Infection: Clinical Features, Treatment, and Nutritional Aspects

**DOI:** 10.3390/diseases9040066

**Published:** 2021-09-23

**Authors:** Merve Öztekin, Birsen Yılmaz, Duygu Ağagündüz, Raffaele Capasso

**Affiliations:** 1 Department of Nutrition and Dietetics, Faculty of Health Sciences, Gazi University, Emek, Ankara 06490, Turkey; megvee.oztekin@gmail.com (M.Ö.); dytbirsen@gmail.com (B.Y.); 2 Department of Nutrition and Dietetics, Faculty of Health Sciences, Çukurova University, Sarıçam, Adana 01330, Turkey; 3 Department of Agricultural Sciences, University of Naples Federico II, 80055 Portici, Italy

**Keywords:** *H. pylori*, nutrition, infection, diet, clinical treatment

## Abstract

*Helicobacter pylori* (*H. pylori*) is a 0.5–1 µm wide, 2–4 µm long, short helical, S-shaped Gram-negative microorganism. It is mostly found in the pyloric region of the stomach and causes chronic gastric infection. It is estimated that these bacteria infect more than half of the world’s population. The mode of transmission and infection of *H. pylori* is still not known exactly, but the faecal–oral and oral–oral routes via water or food consumption are thought to be a very common cause. In the last three decades, research interest has increased regarding the pathogenicity, microbial activity, genetic predisposition, and clinical treatments to understand the severity of gastric atrophy and gastric cancer caused by *H. pylori.* Studies have suggested a relationship between *H. pylori* infection and malabsorption of essential micronutrients, and noted that *H. pylori* infection may affect the prevalence of malnutrition in some risk groups. On the other hand, dietary factors may play a considerably important role in *H. pylori* infection, and it has been reported that an adequate and balanced diet, especially high fruit and vegetable consumption and low processed salty food consumption, has a protective effect against the outcomes of *H. pylori* infection. The present review provides an overview of all aspects of *H. pylori* infection, such as clinical features, treatment, and nutrition.

## 1. Introduction

*Helicobacter pylori* (*H. pylori*) was first identified in the stomach of dogs as a spiral microorganism by Giulio Bizzozero in 1892 [1]. As they are Campylobacter-like spiral microorganisms, they were named *Campylobacter pyloridis* by Barry Marshall and Robin Warren in 1983 [2]. Goodwin et al. named it *“Helicobacter pylori”* in 1989, as it has a helical structure and is mostly found in the pyloric region of the stomach [3]. *H. pylori* is a 0.5–1 µm wide, 2–4 µm long, short helical, S-shaped Gram-negative microorganism and infects more than half of the world’s population [4].

The relationship between *H. pylori* and gastric cancer was investigated in 1991 and 1994, and the International Agency for Research on Cancer, a branch of the World Health Organization, reported that *H. pylori* is carcinogenic in humans, which was reconfirmed in 2009 on the basis of epidemiological data [5,6]. In the United States, the National Institute of Health reported in 1994 that *H. pylori* may be the primary cause of peptic ulcer disease and should be treated. Marshall and Warren were awarded the Nobel Prize in 2005 for their work on *H. pylori* in the field of physiology “for discovering the role of *H. pylori* bacteria in gastritis and peptic ulcer disease” [7]. *H. pylori* plays a role in the development of diseases such as gastritis and mucosa-associated lymphoid tissue (MALT) lymphoma, as well as peptic ulcer and gastric cancer [8].

The mode of transmission of *H. pylori* is not known exactly, but the faecal–oral or oral–oral routes via water or food consumption are thought to be a very common cause [9]. The frequency of *H. pylori* infection increases with age. The rate of development is higher in societies with low socioeconomic status [10]. The fact that *H. pylori* survives in the stomach and creates chronic inflammation shows that it can be resistant to both the immune response and acid [11]. Many antibiotic treatments are used for the treatment of *H. pylori,* and studies show that the number of strains resistant to antibiotics used for treatment is increasing rapidly [12,13], which has led to the search for alternative agents to create safer and more effective results in addition to antibiotic treatments [14].

It is thought that dietary factors may play a considerably important role in *H. pylori* infection, and it has been reported that an adequate and balanced diet, especially high and abundant fruit and vegetable consumption, has a protective effect against the outcomes of *H. pylori* infection [15]. However, some studies have suggested a relationship between *H. pylori* infection and malabsorption of essential micronutrients, and it may cause malnutrition in some groups in the long term [16]. This review aimed to discuss the general clinical features of *H. pylori* and its relationship with nutrition, in addition to the treatment practices related to the disease. 

## 2. *H. pylori* Infection Epidemiology

There are many studies on the prevalence of *H. pylori*, and its risk factors and pathways [9,17,18]. It is claimed that half of the world’s population is infected with *H. pylori*, but it is clear that more evidence-based research is still needed. The incidence of this infection is higher in low socioeconomic status groups and developing countries [19]. Vilaichone et al. found that the prevalence of *H. pylori* varies not only from country to country but also in different regions of the same country [20]. Its prevalence is significantly difficult to determine, as no health system compiles registry-based results of the prevalence of *H. pylori* in developing countries [21].

According to the regional prevalence estimates, there are approximately 4.4 billion *H. pylori*-infected people worldwide [22]. The countries with the highest *H. pylori* burden compared with the general population were found to be Nigeria, Portugal, Estonia, Kazakhstan, and Pakistan, and the lowest burden was in Switzerland [21]. In the study of Mezmale et al. (2020), a high prevalence of *H. pylori* infection was determined in Russia, Jordan, Iran, China, Canada, and Latin American countries [23]. 

Studies conducted in Turkey show that the rate of *H. pylori* infection is high. For example, in a study by Uyanıkoğlu et al. in 2010, 918 of 1298 patients who had antrum biopsy were positive for *H. pylori*. The prevalence of *H. pylori* infection is similar in males and females, and the incidence of *H. pylori* infection is 73.2% between the ages of 14 and 30, 71.5% between the ages of 31 and 45, 68.6% between the ages of 46 and 60, and 70.4% between the ages of 61 and 88 [24]. In a study conducted by Özen et al. in 2011, 161 of 473 children studying in four different primary and secondary schools in Istanbul were found to be *H. pylori-*positive [25]. Similarly, Özaydın et al. screened 4622 people for *H. pylori* infection in 55 cities using the C-urea breath test in 2013, and 3852 people (2075 females and 1777 males) were found to be positive for *H. pylori* [26]. In the review by Hooi et al., it was reported that three studies were conducted in Turkey up to 2015, the total number of participants was 6036, and the prevalence was 77.2% [21]. In a study conducted by Soylu et al. in 2019, the number of *H. pylori*-positive patients was found to be 46 (21 females and 25 males) in biopsy samples taken from 88 patients (53 females and 35 males) aged 18–77 years with dyspeptic complaints. Compared with the total number of participants, male patients were found to be more *H. pylori*-positive [27]. A study conducted in Nepal reported that 18.2% of 6- to 59-month-old children, 14% of boys and 16% of girls aged 10–19 years, and 40% of non-pregnant women aged 20–49 years were infected with *H. pylori* [28].

## 3. *H. pylori* Transmission 

Although the mode of transmission of *H. pylori* is not known exactly, it is thought that it can be transmitted directly from one person to another or indirectly from the environment to people [29]. Person-to-person transmission is thought to be the primary mode of transmission, especially in developed countries. Food- and waterborne transmission are more likely in developing countries and *H. pylori* spreads more rapidly in areas with poor hygienic conditions [30,31].

In a study evaluating the prevalence of *H. pylori* infection in the rural community, Goodman et al. reported that people who are consumers of raw vegetables are more likely to be infected. Moreover, swimming in streams and rivers and using streams as drinking water may increase infection because of contamination by irrigation water or unpurified water [32]. Although some studies suggested that the transmission of *H. pylori* is from environmental contamination to food products, there is insufficient evidence to confirm this information [30,33]. It is accepted that interpersonal transmission routes are more frequent than environmental exposures. However, special attention should be paid to the sources of contamination (unhygienic water) that may lead to contamination through food [29]. 

Person-to-person transmission is thought to occur through the oral–oral, faecal–oral, gastric–oral, or sexual routes [29]. The literature indicates that *H. pylori* is present in the dental plaque and saliva of infected individuals [34,35,36], which shows that *H. pylori* infection spreads at a much higher rate than expected and, especially, transmission between family members is very frequent [37].

## 4. *H. pylori* Diagnosis

Each of the diagnostic tests used to detect the presence of *H. pylori* has advantages, disadvantages, and limitations, and the necessity of endoscopy is taken into account when classifying the methods. Histological evaluations using gastric biopsy specimens include rapid urease testing, culture, and polymerase chain reaction (PCR) [38]. Where invasive methods are time-consuming and not cost-effective, non-invasive diagnostic methods are used. Non-invasive tests include serological evaluation, stool antigen analyses, and the commonly used urea breath tests [39]. On the other hand, there is also non*-Helicobacter pylori helicobacter* (NHPH), which does not have a spiral morphology in the stomach [40]. Neither is the gold standard due to poor sensitivity or specificity. Combinations of more than one test give more reliable results [41].

## 5. *H. pylori* Pathogenesis

*H. pylori* is easily killed in hydrochloric acid solutions with a pH below 4.0. It is quite paradoxical for a microorganism whose primary site is the stomach. *H. pylori* continues to live in the lower part of the stomach by penetrating the mucus layer of the stomach through the contribution of its spiral shape and flagella [42]. To neutralise the acidic pH-related bactericidal activity against *H. pylori*, which can colonise the gastric epithelial surface, *H. pylori* hydrolyses urea to ammonia and carbon dioxide with the urease enzyme it produces [6]. In addition to its toxic effects on gastric mucosal epithelial cells, the ammonia formed increases the mucosal pH [43]. By damaging the protective mucus layer, which is rich in phospholipid and lipase, with the bacterial protease enzyme, it also delays the diffusion ability of H ions and increases its damaging effect [44].

It is known that *H. pylori* secretes a vacuole-forming cytotoxin (VacA) that adheres to the surface epithelium with adhesin proteins and causes vacuolization. The vacuole-forming cytotoxin induces host cell death through pore formation and apoptosis in mitochondrial membranes [45]. In addition to VacA, cytotoxin-associated antigen (CagA), known as an oncoprotein, is delivered into gastric epithelial cells and disrupts vesicular trafficking and autophagy pathways. Various studies have shown that cytotoxin-associated antigens affect the cell shape of bacterial proteins, disrupt cell assembly activity, increase cell motility, and are responsible for gastric ulcers and cancers [46,47,48].

Lipopolysaccharide (LPS), found in the outer membrane of *H. pylori,* is an effective immunomodulator in the human body and causes chronic inflammation by triggering the immune system. LPSs of *H. pylori* can mimic Lewis blood group antigens and, during infection, LPS can produce pathogenic anti-Lewis antibodies [49]. Lewis blood group antigens in the glycoprotein structure found on gastric epithelial surfaces mediate the binding of BabA, known as an adhesin, which binds to blood group antigens on the outer membrane of *H. pylori*, to surface mucosal cells and the gastric pit, and causes tissue destruction [50]. 

## 6. Potential Metabolic Responses to *H. pylori*

Similar to plant and animal species throughout history, humans have been prone to infection by pathogens. It has been suggested that infection formation is associated with many diseases [51]. The gastrointestinal tract constitutes the most intense region in terms of the diversity of microorganisms in the human body, and therefore they have critical roles in the development of the immune system [52]. The stomach was considered a sterile organ unsuitable for the growth of microorganisms. However, the discovery of *H. pylori* has shown that this idea is not correct. With the development of molecular techniques, it has been shown that there are abundant microorganisms in the stomach. In addition, various evidence has indicated that the stomach microbiota is effective in the development and progression of gastric disease [53]. 

The immune response caused by *H. pylori* causes damage to the gastric mucosa. During *H. pylori* infection, surface proteins and LPS are released, stimulating the host’s macrophages and promonocytes [54]. Proinflammatory factors such as interleukin-1 beta (IL-1β), interleukin-8 (IL-8), and reactive oxygen species (ROS) are produced in the gastric mucosa. Moreover, *H. pylori* can interact with epithelial cells to produce IL-8 [55]. IL-1β plays a significant role in the initiation and proliferation of inflammatory responses against bacteria and is effective in the suppression of acid secretion as a key cytokine in the gastric mucosa [56].

*H. pylori* induces the expression of neutrophils and adhesion molecules such as CD11b/CD18 and the production of ROS for potent chemotactic activity by stimulating the secretion of the proinflammatory cytokine IL-8 from the gastric mucosal cells of *H. pylori* [55]. In an in vitro study by Fazeli et al., it was proven that IL-8 is induced by CagA-positive strains of *H. pylori*, causing mucosal damage [57].

Davies et al. have suggested that host neutrophils are involved in the activation of ROS production by *H.pylori* [58]. Excessive ROS production creates oxidative stress in the gastric mucosa and can damage cellular components, including polyunsaturated fatty acids (PUFA), proteins, and DNA [59]. It is thought that *H. pylori* has antigens similar to some humoral compounds that play a role in essential physiological and structural formations in human cells, and that cellular and humoral immune responses can direct tissue destruction towards a pathological inflammatory response [49].

## 7. *H. pylori* and Chronic Gastritis

Inflammation of the gastric epithelium associated with mucosal damage is defined as gastritis [60]. It has been determined that the most common cause of chronic gastritis worldwide is *H. pylori* infection [61]. Proinflammatory cytokine production and inflammation induced by *H. pylori* infection affect gastrin-producing G cells, somatostatin-producing D cells, and acid-producing parietal cells, resulting in significant changes in acid homeostasis in the stomach [62].

Gastritis caused by *H. pylori* also reduces somatostatin levels. Since somatostatin negatively affects gastrin secretion, it causes an increase in gastrin levels and an increase in gastric acid secretion in these patients. Gastrin expression can be enhanced by the direct stimulating effect of *H. pylori*-induced proinflammatory cytokines on G cells [63].

Corpus-dominated gastritis predisposes individuals to gastric cancer, which is partly thought to be due to reduced acid secretion. Infection of the gastric antrum causes increased acid production and predisposes individuals to duodenal ulcer disease, which is associated with a reduced risk of gastric cancer [64]. 

ROS or reactive nitrogen species production is generated by the neutrophils and macrophages/monocytes in response to *H. pylori* infection. These have the potential to cause DNA damage. DNA damage is thought to trigger a series of events in gastric carcinogenesis, represented as a gastritis–atrophy–metaplasia–dysplasia–cancer sequence, by leading to mutations of some important genes in the stomach tissue [65,66]. 

## 8. *H. pylori* and Stomach Cancer

The risk factors of gastric cancer, which is the fifth leading type of cancer worldwide and the third cause of death linked to cancer worldwide, include *H. pylori* infection, age, high salt consumption, and low consumption of fruit and vegetables [67]. 

The pathogenicity of *H. pylori* and bacterial factors, including urease, VacA, CagA, and peptidoglycan outer membrane proteins (BabA, OipA, SabA), affect gastric epithelial cells [68]. Besides, the host’s genetics are affected by *H. pylori* infection, which affects genes encoding cytokines such as IL-8, IL-1β, IL-10, and TNF-α that cause polymorphisms, and increases proinflammatory responses, resulting in gastric cancer risk [69]. Thus, gastric cancer is affected not only by the *H. pylori* strain’s characteristics but also by the host’s genetic determinants and environmental factors [70]. It has also been proven that one of the environmental factors associated with an increased risk of gastric cancer is high dietary salt intake [71].

The noteworthy point in the studies is that a high salt food intake increases CagA levels in *H. pylori* and thus promotes infection [70,72]. The eradication of *H. pylori* may reduce the risk of gastric cancer, and studies have confirmed that it can reduce the occurrence of gastric cancer, including in those at highest risk [73,74]. 

## 9. *H. pylori* and Peptic Ulcers

Peptic ulcer disease, which is a significant source of morbidity and mortality worldwide, usually progresses asymptomatically. Symptoms of symptomatic peptic ulcer disease are epigastric pain associated with bloating, dyspepsia, nausea, early satiety, or abdominal fullness [75]. A peptic ulcer is frequently detected in the stomach and proximal duodenum [76]. Most cases of peptic ulcer disease are thought to be associated with *H. pylori* infection, use of nonsteroidal anti-inflammatory drugs (NSAIDs), or both [77]. 

People with non-atrophic antral-dominant gastritis have high stimulated acid production and increased gastrin levels due to decreased somatostatin in the antrum. Clinically, duodenal ulcers are common in this group [78]. In particular, gastritis caused by *H. pylori* causes a decrease in somatostatin levels [63]. On the other hand, people with atrophic gastritis (concerning both the antrum and corpus mucosa) have impaired acid production. This phenotype is thought to be associated with proximal gastric ulcers, more advanced precancerous lesions, and an increased risk of gastric cancer [79].

Studies have shown improvements in peptic ulcers with the eradication of *H. pylori* [80,81,82]. *H. pylori* chronically colonises the gastric/duodenal mucosa, inducing gastroduodenal diseases such as gastritis and peptic ulcer, and inducing innate and specific immune responses; however, if the infection is not eliminated, the chronic active gastritis condition may continue for life [83].

## 10. *H. pylori* and Anaemia

Iron is an important micronutrient for animals and microorganisms as a cofactor for enzymes involved in oxygen and electron transport and DNA synthesis. The response to infection is mediated by an iron-retaining mechanism that indirectly reduces the redistribution from the cell cytosol to the cell surface, and reduces circulating transferrin and the growth of infecting pathogens [84]. 

Kato et al. demonstrated that the SabA gene in the pathogenesis of *H. pylori* is highly expressed in bacterial isolates from patients with iron deficiency anaemia, proving that this virulence factor has a role in the development of anaemia [85]. Moreover, *H. pylori* causes hypochlorhydria and atrophic gastritis, in addition to peptic ulcer disease and increasing the risk of gastric malignancies. In this case, weakening of iron absorption can cause iron deficiency anaemia [86]. Atrophic corpus gastritis causes impaired intrinsic factor secretion, hypochlorhydria, or achlorhydria and can lead to intestinal iron and B12 malabsorption [86,87]. 

## 11. *H. pylori* and Insulin Resistance

Since the immune system is triggered by *H. pylori* infection, some inflammatory cytokines such as tumour necrosis factor α (TNF-α) and leptin and adipokines create an immune response to this inflammation. Relevant studies have revealed that leptin deficiency can induce the insulin resistance (IR) of high TNF-α and IL-6 levels [88,89]. Inflammatory cytokines induce the phosphorylation of serine residues on the insulin receptor substrate, causing disruption of insulin function and disrupting the substrate’s interaction with insulin receptors. Thus, diabetes can occur with the deterioration in blood glucose regulation [90,91]. 

## 12. *H. pylori* Infection Treatment

Infection treatment is carried out with a combination of antimicrobial agents and antisecretory agents, and gastric pH must be increased with antisecretory agents to achieve the bactericidal effect of antimicrobial agents. Alternatively, herbal medicines and probiotics are used as complementary therapy to help eradicate *H. pylori,* although their mechanism of action is not yet clear [92]. The increasing prevalence of antimicrobial resistance in *H. pylori* from person to person has led to the failure of eradication therapy with decreased compliance with clinical nutrition therapies [93]. 

In the treatment of *H. pylori,* drug resistance can easily develop against antibiotics used alone, so the recommended treatment is a combination of several antibiotics [39]. Many antimicrobial agents, antisecretory agents, and proton pump inhibitors are used in the *H. pylori* treatment protocol, including clarithromycin, amoxicillin, levofloxacin, metronidazole, tetracycline, rifabutin, and bismuth-containing compounds [92,94]. According to several international guidelines, first-line therapy for the treatment of *H. pylori* infection is a triple therapy consisting of a clarithromycin antibiotic given for 7–14 days, using any antibiotic from amoxicillin or metronidazole, and a PPI or ranitidine bismuth citrate [95,96]. If the treatment is not successful, second-line treatment is started. This treatment is carried out according to individual antibiotic resistance and sensitivities, or experimentally [96]. Second-line therapy is usually designated as tetracycline, metronidazole, a bismuth salt, or PPI. After failure of the second-line treatment, antimicrobial susceptibility test should be performed on the *H. pylori* culture from which the gastric biopsy was taken, and local resistance to antibiotics should be taken into account and treatment should be continued [78]. 

It has also been stated that PPIs, which have been used for a long time in the treatment of *H. pylori* infection, may prevent the absorption of micronutrients as well as their benefits [97]. The United States Food and Drug Administration has suggested that long-term use of PPIs may cause an increased risk of hypomagnesemia and fractures [98]. 

## 13. *H. pylori* and Nutrition 

*H. pylori* infection has the main pathogenic effect, especially in diseases of the upper digestive tract. In addition to its cytotoxic and proinflammatory effects, *H. pylori*, similar to other microorganisms in the alimentary tract, affects the brain–gut connection, though indirectly [99]. It produces different biological effects on hormones such as ghrelin and leptin, which control both growth and appetite, causing changes in appetite and food intake, while causing changes in immunological symptoms and responses [99,100].

*H. pylori* is a factor that causes malnutrition and growth retardation, especially in childhood, due to malabsorption of nutrients and increased susceptibility to enteric infections, especially in developing countries [16]. Nweneka et al., in a meta-analysis study, reported that people with positive *H. pylori* had lower circulating ghrelin concentrations in 19 studies. On the other hand, the eradication of *H. pylori* also showed no significant effect on ghrelin in the circulation. This discrepancy in results depends on the amount of damage to ghrelin-producing cells before eradication, the time it takes for these cells to regenerate, and the duration of infection in the circulation [101]. 

Dietary modification to inhibit cancer formation promoted by *H. pylori*, which is a risk factor for cancer, should include eradication as well as practical strategies for the prevention of gastric cancer [102]. In addition, general nutrition has proven to be very important in dietary approaches, as it is known that various nutrients such as vitamin C, iron, cobalamin, and vitamin E cause malabsorption and lead to significant outcomes of nutrition [100].

### 13.1. H. pylori and Salt

*H. pylori* infection, besides being a pathogenic strain, may not be a sufficient reason for the development of gastric cancer. However, the risk for gastric cancer increases with high salt intake [72]. Epidemiological studies have shown a link between high salt consumption and an increased risk of gastric cancer in many parts of the world [103,104].

It has been determined in many studies that high salt intake can affect interactions between the stomach tissue and bacteria, with synergistic effects with *H. pylori*, and increases the possibility of permanent infection [105,106]. CagA, a bacterial oncoprotein, may contribute to the formation of gastric cancer by disrupting the signalling pathways of epithelial cells in the stomach. On the other hand, it has been determined that CagA protein makes an indirect contribution to the pathogenesis of gastric cancer by stimulating increased gastric mucosal inflammation, as it stimulates the proinflammatory cytokine IL-8 and causes an increase in secretion [72].

**Figure 1:**
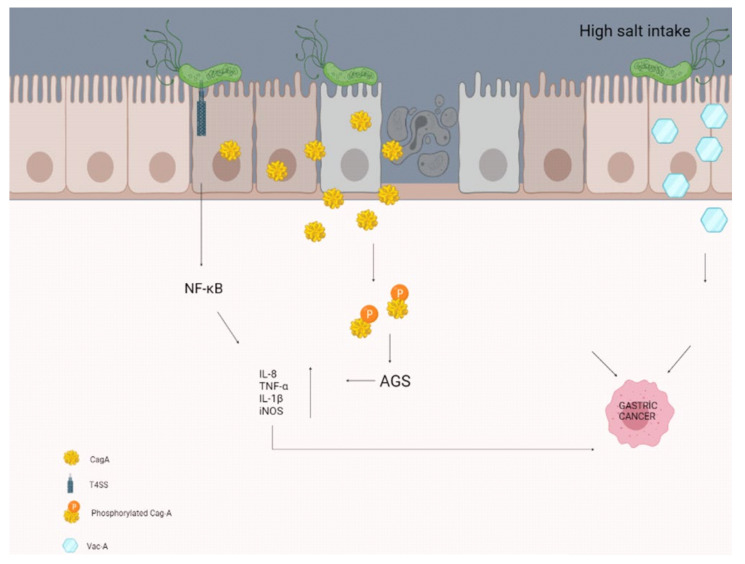
High salt intake may contribute to the formation of gastric cancer by disrupting the molecular pathways and some secretions of the epithelial cells in the stomach.

A study by Gaddy et al. examining the effects of high salt intake with *H. pylori* infection reported that Mongolian gerbils (*H. pylori* infection causes chronic gastritis, gastric ulcer, and intestinal metaplasia in Mongolian gerbils, so it is the best animal model) were infected with a wild-type strain of CagA+ *H. pylori.* Infected animals fed a high-salt diet were reported to have more severe gastroenteritis and a higher rate of gastric adenocarcinoma, with increased expression of the proinflammatory cytokines IL-1β and nitric oxide synthase (iNOS), compared with those fed the normal diet (Figure 1) [107]. Besides, Caston et al. determined an increase in VacA toxin levels in the extracellular space in response to high salt under in vitro conditions. These salt-induced changes have been proven to contribute to an increased risk of gastric cancer in people infected with *H. pylori* who consume a high-salt diet [108].

### 13.2. H. pylori and Iron

Iron is involved in the maintenance of metabolic functions as a cofactor in biological systems that participate in various oxidation–reduction processes, electron transport, and amino acid and nucleotide synthesis, which are essential for life [84,109]. It is also a very important growth factor for almost all bacteria. There is usually a race between bacteria and the host for iron absorption [110]. On the other hand, the interaction of iron with free oxygen creates the Fenton reaction, contributing to the generation of oxygen radicals that can cause serious damage to cellular biomolecules’ structures [111].

**Figure 2:**
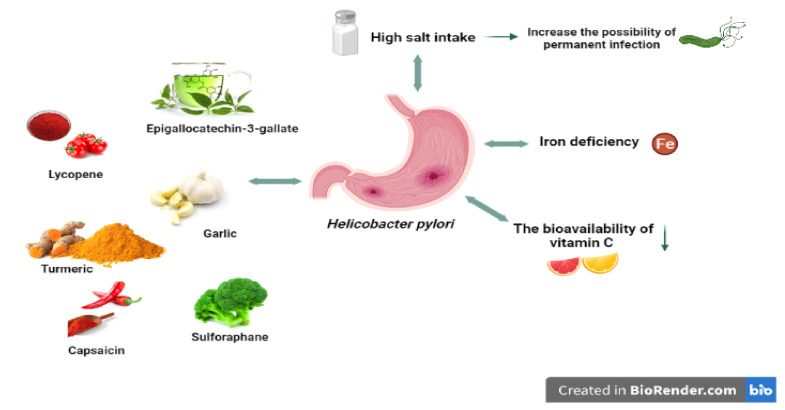
*H. pylori* and related nutritional factors. High salt intake can increase the risk of permanent infection. *H pylori* can cause iron deficiency and decrease the bioavailability of vitamin C. Some nutrients (allicin, lycopene, capsaicin, etc.) have positive effects on *H. pylori*.

*H. pylori*, a pathogen for gastric tissues, undoubtedly encounters a series of environmental stresses in the human stomach it colonises. Iron deficiency is expected in the long term and in some risk groups (Figure 2), as the human body tightly holds the available iron to prevent both bacterial growth and oxidative damage [112]. At the same time, acid secretion due to gastritis caused by *H. pylori* can prevent iron absorption and cause insufficiency [113].

It is thought that iron deficiency, which is a cause of *H. pylori* infection, may also be effective in *H. pylori* virulence (Figure 2) [112,114]. In support of this, an in vitro study found that iron deficiency affected the virulence factors of *H. pylori,* leading to the activity of Cag T4SS and inhibition of gastric acid secretion by the host with increased expression of IL-8, contributing to the increased incidence and severity of gastroenteritis, and thus the development of gastric cancer [115,116]. 

### 13.3. H. pylori and Vitamin C

Vitamin C is a micronutrient essential for human health. Unlike many animals, humans have lost their ability to biosynthesise vitamin C (ascorbic acid) due to various mutations of the gulonolactone oxidase enzyme [117].

Vitamin C is a chemical reducing agent or electron donor. On the other hand, electrons from ascorbate can reduce metals such as iron and copper, leading to the formation of hydrogen peroxide and superoxide and the formation of reactive oxidant species. As a reducing agent, ascorbate can form oxidants in some cases [118]. While vitamin C protects against oxidative stress in cancer cells with its antioxidant effect, it can also increase the risk of cancer with its pro-oxidant activity [119]. 

The bioavailability of vitamin C can be significantly reduced by *H. pylori* infection (Figure 2). In support of this, a study in 1995 showed that *H. pylori* can oxidise and neutralise ascorbic acid in the stomach [120]. In another study, gastric juice and plasma vitamin C levels were significantly reduced with decreased vitamin C intake in people with *H. pylori* [121]. In such studies, it is expected that vitamin C levels will return to normal after *H. pylori* eradication [122,123]. Atrophic gastritis, which is a result of *H. pylori* and triggers the formation of gastric cancer, may disrupt the secretion of vitamin C in the gastric mucosa and increase the pH of gastric juice [124].

In a study conducted by Waring et al., when a control group consisting of 48 gastritis patients without supplementation and an experimental group of 32 gastritis patients who were given 500 mg vitamin C twice a day for 2 weeks were compared, it was found that high vitamin C intake could reduce the risk of gastric cancer, but showed that the protective effect may be greater if gastritis is treated with *H. pylori* eradication [125]. In another study, Zojaji et al. divided the study group into two, created the same treatment protocol for both groups, and added vitamin C to the second group. The experimental results showed that 78.0% of individuals in the group receiving supplemental vitamin C had increased *H. pylori* eradication rates compared with 48.8% of individuals in the other group [126]. 

On the other hand, side effects such as gastrointestinal disturbances and especially osmotic diarrhoea may occur due to high doses of vitamin C. It also causes an increase in uric acid and oxalate excretion, and may also be associated with an increased risk of kidney stones caused by calcium oxalate [127]. In rare cases, due to its pro-oxidant activity at high concentrations, especially in supplement form, high concentrations (500 mg/day or more) are expected to cause various adverse effects such as DNA damage and, indirectly, some types of cancer in the presence of high iron stores in the human organism [128]. Given that it is a water-soluble vitamin, alongside the chance of toxicity from a single daily bolus dose and its short biological half-life, more clinical research is required to determine the correct dosage [129]. However, vitamin C, which is generally taken with natural nutrition, increases mucosal immune responses by eliminating free radicals, especially through fruit and vegetable consumption [130]. It reduces the content of N-nitrosamine in the gastric juice and inhibits cell proliferation. It may especially be protective against gastric carcinogenesis associated with *H. pylori* by directly affecting the growth of *H. pylori* [131].

### 13.4. H. pylori and Antioxidants

Oxidative stress is a physiological process experienced by every living organism and plays a role in the aetiology of many diseases and the ageing process [132]. The stomach, which is a bioreactor, is an excellent environment to increase the co-oxidation of vitamins but is constantly exposed to reactive species and ingested carcinogens, bacterial pathogens, and oxidative compounds associated with food digestion [132,133]. 

After *H. pylori* reaches the gastric epithelium, it activates nicotinamide adenine dinucleotide phosphate hydrogen (NADPH) oxidase and produces ROS such as hydrogen peroxide (H_2_O_2_), superoxide (O_2_-), hypochlorous acid (HOCl), and hydroxyl radicals (OH) in gastric epithelial cells independently of inflammatory cells, thus triggering infection responses in the target host’s innate immune cells [134,135]. 

ROS production by *H. pylori* activates the oxidant-sensitive transcription factor NF-κB, which induces the expression of oncogenes and cell cycle regulators and plays an important role in apoptosis and DNA damage in gastric epithelial cells [136]. It was observed that ROS activity increased and antioxidant compounds such as vitamin C decreased in *H. pylori*-positive individuals. Thus, gastric cancer develops due to the DNA damage caused by *H. pylori* [137]. It is also known that *H. pylori* infection disrupts the function of some oncogenes and tumour suppressor genes (e.g., p53) in gastric tissue, and this may trigger the initial carcinogens [138,139].

Since *H. pylori* infection affects the oxidative stress process, it is thought that some antioxidant foods and nutrients may be effective. This is also emphasised in the literature investigating the relationship between *H. pylori* infection and nutrients, which suggests that the inclusion of these nutrients in the diet may have a protective effect, unlike some other nutritional components such as salt (Figure 2).

Garlic (*Allium sativum L*.), a member of the lily family, known worldwide and most widely cultivated in Asia, is a medicinal plant. It contains 33 sulphur compounds, especially allicin. Garlic is known as an effective free radical scavenger against various diseases caused by ROS [140]. Chung et al. showed in 1998 for the first time that garlic components can suppress the growth of *H. pylori* [141]. Zardast et al. argued that garlic had antibacterial effects against *H. pylori* in *H. pylori*-positive individuals who consumed two medium raw cloves of garlic (3 g) with daily meals twice a day for 3 days [142]. In the suppression of *H. pylori*, the allicin in garlic inhibits the activation of NF-κB by inhibiting the Toll-like receptor 4 (TLR4) signalling pathway, resulting in an anti-inflammatory effect [143].

Turmeric (*Curcuma longa*) contains curcumin, a polyphenolic compound, and has a yellow pigment. It is widely used as a food colouring agent [144]. Curcumin has a wide range of beneficial properties, including antioxidant, anti-inflammatory, anticancer, antiproliferative, antifungal, and antimicrobial properties [145]. Judaki et al. formed two groups of *H. pylori*-positive individuals and applied the same treatment protocol to both groups by adding 700 mg of oral curcumin three times a day to the second group. After 3 months, they reported a significant reduction in the amount of oxidative DNA damage in the curcumin group [146].

Lycopene, a natural antioxidant, is a carotenoid that gives fruits and vegetables their red colour [147]. Some studies revealed that lycopene inhibits DNA damage and the cellular response of *H. pylori*-infected gastric carcinoma cells (AGS) [148,149]. In a study by Shidfar et al., it was shown that the effect of lycopene was not significant in the treatment of *H. pylori-*positive patients [150]. The main findings of some dietary interventions in *H. pylori* are summarised in Table 1.

Pepper, a member of the *Solanaceae* family, has high nutritional value in terms of antioxidant properties, as it is a rich source of carotenoids and provitamin A, in addition to vitamins E and C [153]. Capsaicin, the active component of hot peppers, gives peppers a characteristic pungent flavour [154]. It was thought that capsaicin might have a protective effect against *H. pylori* infection, and in 1997, Jones et al. showed that capsaicin produced effective inhibition of *H. pylori* growth in vitro, depending on the time and concentration [155]. Capsaicin has also been suggested as a potential anti-inflammatory drug by inhibiting IL-8 production in the gastric epithelium of *H. pylori-*positive individuals [156]. More research is needed to better understand its effects and to determine the amount for daily consumption.

Epigallocatechin-3-gallate (EGCg) is the major and the most significant polyphenol found in green tea and is effective in health as an anti-inflammatory, antioxidant, and anti-atherogenic agent [157]. It was thought that it might be effective against infection caused by especially resistant *H. pylori* strains [158]. An in vitro study by Yanagawa et al. revealed that the addition of EGCg to antibiotic treatments resulted in increased antibacterial activity and showed a relatively slow but strong activity against the growth of *H. pylori* [151]. 

Another structure with bactericidal properties against *H. pylori* infection, which contributes to the formation of stomach cancer, is sulforaphane. It is abundant in broccoli sprouts and has a protective effect on injuries caused by various types of oxidative stress thanks to its phytochemicals and antioxidant enzymes [159]. Sulforaphane not only increases the antioxidant activity of the gastrointestinal mucosa but also inhibits the growth of various microorganisms, including some human pathogens, and has anticarcinogenic activity [160,161]. *H. pylori* infection in the gastric mucosa induces oxidative stress. It is known that genes encoding nrf2 (NF-E2 p45-related factor-2) and keap1 (Kelch-like ECH-related protein 1) play a critical role in the activities of antioxidant enzymes. Sulforaphane stimulates antioxidant enzyme activities linked to the nrf2 gene, thus enabling *H. pylori* to inhibit oxidative damage in cells [152].

In a study by Yanaka et al., people who were *H. pylori*-positive were fed broccoli sprouts (70 g/day) and compared with the placebo group. People who consumed broccoli sprouts for 8 weeks showed positive effects in the clinical laboratory results. However, a regression to the starting point shortly after the end of his study was observed [162]. Therefore, more studies are needed to determine its long-term effects.

### 13.5. H. pylori, Nitrite, and Nitrosamines

Nitrosamines are formed by the reaction of secondary amines with nitrites. N-nitroso compounds (NOCs) and nitrosamines contribute to gastric carcinogenesis. They can be formed exogenously (during fermentation, smoking, cooking, and storage) or endogenously in the stomach unless inhibited by vitamin C or other antioxidants [163,164]. The nitrite-clearing ability of vitamin C depends on the stomach pH and the vitamin C/nitrite ratio, so it causes an increase in NOCs in the case of pH>4 and a decrease in the vitamin C/nitrite ratio [165]. 

*H. pylori* stimulates the macrophage system via the L-arginine/NO pathway [166]. It is thought that *H. pylori* infection will not only trigger the formation of NO endogenously but also cause DNA damage and increase the risk of cancer [166,167]. On the other hand, various foods such as pickled foods and processed meat, which are sources of nitrites and/or nitrosamines, are also important sources of salt [168]. In general, *H. pylori*-positive individuals with a high dietary salt intake have an increased risk of gastric cancer compared with those who are *H. pylori*-negative and with low-salt intake, and red meat-related gastric cancer risk, endogenous nitrosamine formation, or consumption of processed meat is only more common in *H. pylori*-positive individuals [169]. 

### 13.6. H. pylori and Probiotics

The term “probiotic” is of Greek origin and is translated into our language as “pro bios” (pro: for, bios: life) meaning “for life” [170]. According to the publication of the International Scientific Association of Probiotics and Prebiotics (ISAPP) in 2021, probiotics are defined as live microorganisms that, when taken in sufficient amounts, provide health benefits to the host [171].

Probiotic bacteria can bind to recognition receptors, such as TLR expressed on the surface of epithelial cells, thereby triggering several immunological defence mechanisms. It has been reported that probiotics can modify the immunological response by increasing the production of anti-inflammatory cytokine-regulating (IL-10 and TGF-b) cells and/or suppressing cytokines (IL-4, IL-5, IL-6, and especially IL-8), thus reducing gastric activity and inflammation, that is, the effect of *H. pylori* infection [172]. 

In the Maastricht-5 Consensus Report, it was stated that the stomach microbiota decreased with the use of various antibiotics and it was accepted that probiotics were beneficial, but the level of evidence was evaluated as moderate with a low recommendation [78]. Additionally, the use of adjuvant probiotic treatments (*Lactobacillus* spp. and *Saccharomyces boulardi*) in combination with antibiotics may be beneficial [78]. In patients with *H. pylori* infection, *Lactobacillus* spp. supplementation in addition to triple therapy may be beneficial in improving *H. pylori* eradication rates, especially in children, and in reducing treatment-related side effects (especially the incidence of diarrhoea) [173,174]. The first meta-analysis study conducted in 2010 on the use of *Saccharomyces boulardi* as an adjuvant therapy found that it generally reduced the side effects of *H. pylori* infection [175]. *Saccharomyces boulardi* supplementation along with the standard triple therapy provided additional support to increase *H. pylori* eradication rates [176]. 

To sum up, it has been reported that some probiotics reduce the side effects of infection in the treatment to eradicate *H. pylori*. More clinical research is needed to gain insight into the direct efficacy of specific probiotic strains, including the duration and dosages of adjuvant probiotic therapy, the individual’s lifestyle (such as alcohol, diet, or cigarette consumption), and geographic differences [132]. 

## 14. Conclusions

*H. pylori* is estimated to infect half of the world’s population and causes permanent infections as well as many health issues such as gastritis and MALT lymphoma, as well as peptic ulcer and gastric cancer. In *H. pylori* infection, there are some treatment limitations due to its ability to create resistance to antibiotic treatments in treatment strategies. Therefore, it has become necessary to seek alternatives to fight against *H. pylori* infection. Especially in the last few years, research has clearly shown the pathogenicity, microbial activity, and genetic predisposition to help understand the severity of gastric atrophy and gastric cancer caused by *H. pylori*. This situation is expected to affect the treatment process positively. Combination treatments, including with phytochemicals and probiotics found in natural products, seem to have beneficial effects in the eradication of *H. pylori.*

Due to the effects of hormones such as ghrelin and leptin, which control both growth and appetite, and the formation of malabsorption of various nutrients such as vitamin C, iron, cobalamin, and vitamin E in *H. pylori*-infected individuals, detailed nutritional information should be provided during and after treatment. It is important to provide optimal nutrition through the determination of strategies and the application of a suitable diet for the person by authorised dietitians. Besides, there have been some promising effects for probiotics added to treatment strategies; however, detailed research is needed. Most importantly, a diet rich in fruits and vegetables and reduced in salt and processed meat products has good prophylactic potential, especially against cancer in the eradication of *H. pylori*.

## Figures and Tables

**Table 1:** The main findings of dietary interventions in *H. pylori.*

Dietary Intervention	Study Design	Main Outcomes	Reference
High-salt diet	*Mongolian gerbils were infected with a wild-type (WT) CagA(+) *H. pylori* strain or an isogenic CcagA mutant strain. A regular diet or a high-salt diet was given to the animals.	Among animals infected with the WT strain, those fed a high-salt diet had more severe gastric inflammation, higher gastric pH, increased parietal cell loss, increased gastric expression of interleukin 1β (IL-1β), and decreased gastric expression of hepcidin and hydrogen potassium ATPase (H, K-ATPase) compared with those fed a regular diet.	[107]
Vitamin C	32 patients were supplemented with ascorbic acid (500 mg twice daily for 2 weeks) 48 patients were not supplemented	High ascorbic acid may lower the incidence of gastric cancer, although its protective impact may be enhanced if gastritis is addressed (for example, by *H. pylori* eradication).	[125]
Vitamin C	Patients with *H. pylori* were randomly divided into two groups; -Group A (n: 162) were given a treatment regimen -Group B (n: 150) received the same regimen + 500 mg of vitamin C/day	The *H. pylori* treatment regimen with vitamin C may significantly increase the *H. pylori* eradication rate.	[126]
Garlic	15 patients with *H. pylori* During 3 days, two medium-sized cloves of garlic (3 g) were given twice/day with meals.	Raw garlic has antibacterial effects against *H. pylori* residing in the stomach. It may be prescribed with drugs in the treatment of *H. pylori* infection.	[142]
Curcumin	Two groups of *H. pylori*-positive patients The same treatment protocol was applied to both groups 700 mg of oral curcumin (three times/day) was added to the second group.	Curcumin may be a useful supplement to improve chronic inflammation as well as preventing carcinogenic changes associated with *H. pylori* in patients with chronic gastritis.	[146]
Lycopene	54 *H. pylori*-positive patients; Four standard drugs were given to Group 1. Lycopene (30 mg/day) was given to Group 2.	Lycopene does not have any significant effects on eradicating *H. pylori* when compared with the standard antibiotic therapy.	[150]
EGCG	56 clinical isolates of *H. pylori*, including 19 isolates highly resistant to metronidazole (MTZ) and/or clarithromycin (CLR), were used to determine in vitro sensitivity to tea catechins.	EGCG may be a valuable therapeutic agent against *H. pylori* infection.	[151]
Sulforaphane	48 *H. pylori*-infected patients were randomly divided into two groups: Broccoli sprouts (70 g/day, containing 420 micromoles of sulforaphane precursor) for 8 weeks Placebo—consumption of an equal weight of alfalfa sprouts (not containing sulforaphane)	The treatment seemed to enhance chemoprotection of the gastric mucosa against *H. pylori*-induced oxidative stress.	[152]
